# BioInfer: a corpus for information extraction in the biomedical domain

**DOI:** 10.1186/1471-2105-8-50

**Published:** 2007-02-09

**Authors:** Sampo Pyysalo, Filip Ginter, Juho Heimonen, Jari Björne, Jorma Boberg, Jouni Järvinen, Tapio Salakoski

**Affiliations:** 1 Turku Centre for Computer Science (TUCS), and the Department of IT, University of Turku, Lemminkäisenkatu 14a, 20520 Turku, Finland

## Abstract

**Background:**

Lately, there has been a great interest in the application of information extraction methods to the biomedical domain, in particular, to the extraction of relationships of genes, proteins, and RNA from scientific publications. The development and evaluation of such methods requires annotated domain corpora.

**Results:**

We present BioInfer (Bio Information Extraction Resource), a new public resource providing an annotated corpus of biomedical English. We describe an annotation scheme capturing named entities and their relationships along with a dependency analysis of sentence syntax. We further present ontologies defining the types of entities and relationships annotated in the corpus. Currently, the corpus contains 1100 sentences from abstracts of biomedical research articles annotated for relationships, named entities, as well as syntactic dependencies. Supporting software is provided with the corpus. The corpus is unique in the domain in combining these annotation types for a single set of sentences, and in the level of detail of the relationship annotation.

**Conclusion:**

We introduce a corpus targeted at protein, gene, and RNA relationships which serves as a resource for the development of information extraction systems and their components such as parsers and domain analyzers. The corpus will be maintained and further developed with a current version being available at .

## Background

Recent advances in biomedical research methods have greatly accelerated the rate at which new information is published. As a result, there has been an increased interest in applying Natural Language Processing (NLP) methods to the domain of biomedical publications accessible in literature databases such as PubMed [1-4]. The attention of the BioNLP community has recently focused on Information Extraction (IE), in particular the development of IE systems for extracting protein-protein interactions.

Information extraction systems automatically identify entities and their relationships from free text, producing a structured representation of the relevant information stated in the input text. Such systems can, for example, support researchers in literature searches and serve as the basis for the inference of semantic relationships, such as candidate pathways, stated across several publications.

An annotated corpus is a collection of texts that have been enhanced with markup specifying linguistic and domain information such as syntactic structure, named entity identification, and entity relationships. In this paper, we introduce BioInfer (Bio Information Extraction Resource), a manually annotated corpus resource for IE method evaluation and development in the biomedical domain, accompanied by supporting software. The corpus consists of 1100 sentences and represents 15 man-months of annotation efforts. We further present an annotation scheme that combines key annotation types with a detailed definition of entity relationships as well as present two new biomedical ontologies that define bioentity and relationship types.

The availability of annotated corpora is an important prerequisite for NLP research as they provide gold-standard data for method evaluation and development. The impact of the MUC, TREC and SENSEVAL conferences [5-7], for example, has shown how a common shared corpus resource can result in increased focus and rapid advances in the field. Due to the different nature of the language used in biomedical scientific publications, the existing standard corpora of, for example, general and newspaper English are only of limited utility. With the increased interest in biomedical NLP research, the need has thus emerged for corpora that specifically target the biomedical domain.

Most important among the steps typically applied by IE systems to extract information from text are named entity recognition, parsing and domain analysis, where named entity recognition identifies the entities whose relationships are to be found, parsing recovers the syntactic structure of the text, and domain analysis extracts the relationships among the named entities using the information from the other processing steps. Several biomedical corpora have been developed to facilitate the development of the separate IE system components, providing, for example, bioentity, entity relationship, syntax, abbreviation, and coreference annotation – we discuss these corpora and relate them to BioInfer in the Results and Discussion section. By contrast, the BioInfer corpus provides all these key types of annotation together, for a single set of sentences. By providing gold-standard input for the major stages in IE, the corpus allows the parallel development of all IE system components. The shared data facilitates the study of the interplay of the components, for example allowing the source and propagation of errors to be analyzed.

Additionally, the corpus addresses a number of issues in the prevailing relationship annotation approach, in which relationships are expressed as pairs of entities. The BioInfer annotation allows relationships with a complex structure, such as relationships between relationships or relationships of more than two entities. Moreover, the BioInfer annotation scheme defines entity and relationship types that are organized into two interdependent hierarchical ontologies. The entity type ontology incorporates the established Genia ontology of physical types [8]. For the users of the corpus, the ontologies precisely define which types of entities and relationships are annotated and how they are related. By binding the corpus text to typed entities and relationships, the annotation also provides a mapping from the open domain of natural language statements to a limited, controlled vocabulary of types in the ontologies, specifying the words that are used to state entities and relationships of each type. In applications of the corpus, the ontologies can be used to view the annotation at different levels of abstraction as well as serve as a basis for further interpretation and normalization of the annotated entities and relationships. The syntactic annotation of the corpus follows the Link Grammar dependency formalism [9]. Additionally, it is designed to facilitate automatic transformation to other dependency formalisms.

In previous studies, we have used an early subset of the corpus syntactic annotation to evaluate the performance of the Link Grammar and Connexor Machinese Syntax dependency parsers in the biomedical domain [10,11]. The annotation allowed a detailed error analysis which identified a number of areas for future domain adaptation of Link Grammar, such as that performed by Aubin et al. [12]. For the analysis of Connexor Machinese Syntax, we found that while 26% of the corpus dependencies had to be modified to match the Connexor scheme, the corpus annotation allowed most of these to be handled automatically, leaving only 6% of all dependencies to be manually changed. This demonstrates the applicability of the corpus also to the evaluation of dependency parsers other than Link Grammar. The corpus has also been used as reference data in performing lexical adaptation of Link Grammar Parser to the biomedical domain, showing statistically significant improvement in performance for many adaptation approaches over the performance of the unmodified parser [13]. The corpus can further be used for many machine learning tasks. For example, we have used the corpus syntactic annotation as training and evaluation data in developing kernel-based machine learning methods for parse reranking. These ranking methods were shown to significantly outperform the Link Grammar Parser ranking heuristics, leading to improved parsing performance [14-16].

The entity relationship annotation brings insight into the various relationship types that can hold among entities in the text. It further reveals the often complicated structure of these relationships and the entities themselves. The primary use of the annotation is to develop and test an IE system that targets the relationships stated in the text. Due to the fact that both the dependency and the relationship annotation cover the same set of sentences, the interplay between the syntax and the relationships can be studied as well. Further, as both the entity and relationship types are organized in hierarchical ontologies, the annotation facilitates easy step-by-step development whereby only certain types of relationships and certain types of entities are addressed at once.

The paper is organized as follows. We first briefly introduce the corpus annotation and state the main results. We then describe the main features of the relationship and entity annotations, the ontologies used for defining the types of relationships and entities, and the binding of these annotations to text of the corpus sentences. We then present key features of the dependency annotation and finally present corpus statistics and supporting software, and discuss the relationship of the BioInfer corpus to other biomedical corpora. In the Methods section, we discuss in further detail the three main annotation types, presenting, for example, the annotation for anaphora and abbreviation definitions.

## Results and discussion

The corpus annotation is divided into three key types, termed *entity annotation, entity relationship annotation*, and *dependency annotation*. We now briefly introduce these annotation types; more detailed descriptions are given in the Corpus annotation section.

The foundation of the entity annotation is the identification of *named entities *of the gene, protein, and RNA types. When relevant to relationships, other physical entities as well as abstract process and property entities pertaining to the named entities are also identified in the annotation. For example, in the sentence

*Deletion of SIR4 enhanced METI5 silencing*.

the annotated entities are not only the genes *SIR4 *and *METI5*, but also the processes *deletion of SIR4 *and *METI5 silencing *that pertain to the genes. Together with entity typing, this extended annotation allows a detailed resolution of the relationships stated in the sentence.

The entity relationship annotation describes relationships holding between the entities as stated in the individual sentences. The relationships are annotated through logic formulas where the predicates define relationship types and predicate arguments identify the entities that are related. For example, the relationships of the preceding example sentence are annotated as

*STIMULATE*(*deletion of SIR4*, *METI5 silencing*)

Note that the relationship stated with the word *enhanced *is expressed using the predicate *STIMULATE*. Throughout the text, we follow the convention that predicate names are capitalized. The relationship annotation scheme is also used to annotate abbreviation definitions, and, where necessary to extract relationships, also coreference through, for example, pronouns.

**Figure 1:**
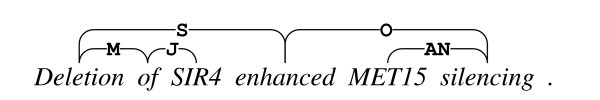
**Dependency annotation example**. The link labels represent types of dependency, for example, S for subject-predicate and O for predicate-object dependency.

Finally, the dependency annotation describes the syntax of each sentence in the corpus. Each sentence is given a full dependency syntax annotation based on the Link Grammar dependency syntax formalism. An example of the dependency annotation is given in Figure 1.

**Figure 2:**
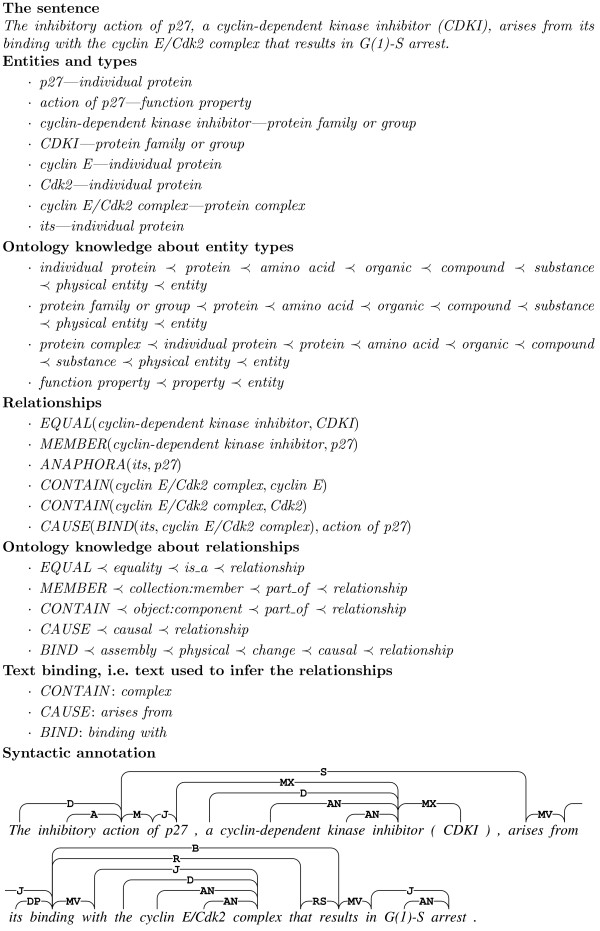
**Illustration of corpus annotation and ontology information**. The ≺ symbol stands for the *is a *relationship between ontology terms.

Figure 2 illustrates a complete sentence with all the information given by the corpus annotation and the ontologies.

We now briefly summarize our contribution. The primary outcome of the research presented in this paper is the corpus itself, a public resource that facilitates the development of IE systems in the biomedical domain, available along with supporting software in a format that is straightforward to process [17]. The corpus contributes hand-annotated gold-standard data to a domain where such resources are scarce. Further, the BioInfer corpus is unique in combining the named entity, syntax, and entity relationship annotations for the same set of sentences and in the level of detail of the relationship annotation.

Another outcome of the research are the ontologies, in particular the relationship type ontology, which defines a large number of possible relationship types and organizes them in a hierarchical manner. The ontologies can be used to focus an IE system only to certain types of relationships and entities, possibly considering different levels of generality. The ontologies can also be used to support various automated reinterpretations of the annotation based on rules attached to the various entity and relationship types in the ontology. Moreover, the binding of entities and relationships stated in the corpus text to specific types in the two ontologies defines mappings from natural language statements to controlled vocabularies, providing data for the analysis of how each concept is expressed in practice.

The process of designing the annotation scheme and building the corpus has further contributed to a better understanding of the domain of entity relationships. An analysis of the corpus shows that 10% of the relationships are of a complex nature with more than two related entities and relationships affecting other relationships rather than only entities. Further, 14% of the entities involved in a relationship are processes or properties as opposed to physical entities. These results illustrate the information loss in the prevailing annotation approach where only pairwise relationships are used to relate only physical entities.

Moreover, we find that there is no one-to-one correspondence between relationship types and the words that state the relationship in the text. Many expressions are ambiguous, that is, they can be used to state different relationship types, depending on the context. This emphasizes that context-based disambiguation is necessary to resolve relationship types in the text.

### Corpus annotation

The BioInfer corpus consists of individual sentences, each of which is separately assigned the entity, relationship, and dependency annotations. For example, in the sentence

*Interaction of Munc-18-2 with syntaxin 3 controls the association of apical SNAREs in epithelial cells*.

we find four entities: three proteins (*Munc-18-2*, *syntaxin 3*, *SNAREs*) and a process related to a protein (*association of SNAREs*). Further, there are two relationships: the *interact *relationship between the *Munc-18-2 *and *syntaxin 3 *proteins and the *control *relationship between the previous *interact *relationship and the *association of SNAREs *process. The dependency syntax specifies, for example, a subject-predicate dependency between the words *interaction *and *controls *and a object-predicate dependency between the words *association *and *controls*. There is an interdependence between the various annotation types in the sense that, for example, the dependency annotation respects named entities and, conversely, certain rules for annotating the entities and relationships make use of the syntax. For the purpose of this presentation, we will, however, treat the annotation types separately.

#### Entity relationship annotation

The entity relationship annotation, or relationship annotation in short, is based on the notions of an *entity *and a *relationship*. An entity denotes a named bioentity or a physical or abstract entity pertaining to a named bioentity. A relationship captures a stated relation between two or more entities or other relationships. Both the entities and the relationships reflect the information explicitly stated in the sentence. They exist in their own right, abstracted from the sentence text, and we first discuss them on this level. Later in this section, we introduce how the entities and relationships are bound to the actual text of the sentences.

The relationships belong to a variety of *relationship types *and are often structurally complex. The relationship types can be, for example, *observed co-occurrence, sequence similarity*, or *physical binding*. For example, the two relationships (*bind*, *promote*) in the sentence

*mDia binds profilin to promote actin polymerization*.

are of distinctly different types. Further, a relationship can be stated on different levels of specificity. In the sentence

*Aip1p interacts with cofilin to disassemble actin filaments*.

there are two relationships: *interact *and *disassemble*. Clearly, *disassemble *is a more specific statement than *interact*.

In order to capture the various relationship types as well as their different levels of specificity, we introduce the relationship type ontology that defines relationship types and organizes them in a hierarchical manner, from the most general to the most specific relationship types. The ontology specifies over 60 relationship types at 5 levels of generality. Each annotated relationship is categorized within this ontology at the most specific level applicable.

Apart from belonging to a large variety of types, the relationships are also structurally complex. The prevailing approach in previously published biomedical corpora has been to only annotate pairwise relationships between physical entities. However, it becomes clear that this representation in many cases fails to accurately capture the stated information. Consider, for example, the following sentence.

*Four yeast spliceosomal proteins (PRP5, PRP9, PRP11, and PRP21) interact to promote U2 snRNP binding to pre-mRNA*.

Using only pairwise relationships, the sentence would most likely be decomposed into all pairwise relationships among the spliceosomal proteins and all pairwise relationships between the spliceosomal proteins and *U2 snRNP*. However, any such decomposition leads to significant imprecisions. The sentence does not specify which pairwise interactions take place among the four spliceosomal proteins, nor does it assert a pairwise relationship between any of the *spliceosomal proteins *and, for example, *U2 snRNP*. Instead of using only pairwise relationships, the information in the sentence can be better captured through structurally more complex relationships: an *interact *relationship among all the four spliceosomal proteins and a *promote *relationship between the *interact *relationship and the pairwise *bind *relationship. In the BioInfer corpus, the relationships are annotated using logic formulas. Each relationship is expressed through a predicate whose arguments are instantiated with entities or other predicates. The predicate name, arity, and the semantic roles of its arguments are defined in the relationship type ontology together with a description of the relationships intended to be annotated using this predicate. The relationship annotation of a sentence is then a set of formulas that state all the relevant relationships in the sentence. Let us consider the previous example sentence. The formula

*INTERACT*(*PRP5*, *PRP9*, *PRP11*, *PRP21*)

expresses the relationship among the spliceosomal proteins. In the usual interpretation, it does not explicitly assert a pairwise relationship between any two of the four proteins. The formula

*STIMULATE*(*INTERACT*(*PRP5*, *PRP9*, *PRP11*, *PRP21*), *BIND*(*U2 snRNP*, *pre-mRNA*))

describes the relationships stated in the sentence, accounting for their complex nature. It is this formula that is used in the corpus to annotate the example sentence.

The formula-based annotation system is more powerful than the simple pairwise relationship annotation. However, practical considerations and the current state-of-the-art in IE systems often call for pairwise relationships, even at the cost of introducing inaccuracies such as those discussed above. It is thus necessary to employ a scheme where the annotation can be decomposed in terms of pairwise relationships. The BioInfer annotation scheme allows such decomposition. Multiple-argument predicates such as *INTERACT*(*PRP5*, *PRP9*, *PRP11*, *PRP21*) can be decomposed in the obvious manner and predicates whose arguments are other predicates can be either omitted or recursively decomposed. It depends on the predicate whether better approximation can be achieved by omission or by recursive decomposition. Several examples of the relationship annotation are provided in Appendix I.

#### Entity annotation

The BioInfer corpus is focused on the development of IE systems for extracting relationships between genes, proteins, and RNAs. This focus influences the entity annotation as currently only entities that are relevant to this focus have been annotated. As a typical IE system extracts relationships between named entities, we require that an entity is named or pertaining to a named entity, more specifically a named gene, protein, or RNA, in order to be relevant to the corpus focus. For example, *actin *is a named entity, *actin expression *pertains to a named entity, but *a 50 kDa protein *as an entity is not considered named and thus not annotated in the current version of the corpus. The typical use in literature and databases such as Swiss-Prot is used as a guide in deciding for borderline cases whether an entity is considered named or not named.

In addition to named entities, *extended named entities*, defined as terms denoting other physical entities, processes and properties pertaining to named entities, are also annotated when relevant to relationships. Consider the following example:

*Deletion of SIR4 enhanced mURA3 and METI5 silencing*.

The sentence contains three named entities (*SIR4*, *mURA3*, *MET15*). Further, the entity *deletion of SIR4 *is a process extended named entity pertaining to *SIR4*, similarly for *mURA3 silencing *and *METI5 silencing*. In the sentence

*Finally, both receptors can interact with FADD, TRADD, and RIP*.

the annotation identifies three entities: the named entities *FADD, TRADD*, and *RIP*. The reference to *both receptors *does not constitute an entity, because it is not a named entity, nor it is pertaining to a named entity (within the sentence).

While it is common to restrict IE systems to only take into account named bioentities as objects participating in relationships, consideration of extended named entities allows a better resolution of the actual statements in the sentence. Consider the following example:

*Abundance of actin is affected by the differential calreticulin expression*.

Although it is not incorrect to state that *calreticulin *affects *actin*, a more detailed resolution of the entities is desirable. The *affect *relationship in this sentence is between two property/process entities: *abundance of actin *and *calreticulin expression. Abundance of actin *is a property pertaining to *actin *and *calreticulin expression *is a process pertaining to *calreticulin*. Both *actin *and *calreticulin *are named entities. As a further illustration, consider the sentence

*Characterization of gelsolin truncates that inhibit actin depolymerization by severing activity of gelsolin and cofilin*.

where the related entities are *gelsolin truncates, actin depolymerization, activity of gelsolin *and *activity of cofilin *rather than the proteins *gelsolin, actin*, and *cofilin *themselves.

The need to resolve that, for instance, *abundance of actin *is a protein amount property pertaining to the protein *actin *leads to two crucial entity annotation concepts: *entity nesting *and *entity typing*. Entity nesting allows annotation of entities within entities with the outer entity typically being a semantic modification, extension or specification of the inner entity. For instance, the outer entity *abundance of actin *is a specification of the inner entity *actin *in the sense that it identifies a single property of actin. Each entity is given a type from the hierarchical ontology of entity types developed for this purpose. The ontology captures all relevant entity types, both physical entity types such as *gene *and *protein *as well as abstract entity types such as *process *and *property*. Similarly to the relationship type ontology, the entity types are organized in a hierarchical manner and in the annotation each entity is given the most specific applicable type from the ontology.

The introduction of entity nesting and typing allows us now to state a general principle regarding the entity annotation. Only named entities of the *gene, protein*, and *RNA *types are annotated in each sentence. In addition, those named entities that participate in a relationship are nested inside the broadest entity relevant to the particular relationship. Thus, up to special cases described later, every entity that participates in an annotated relationship has a named gene, protein or RNA at its innermost level of nesting.

This is demonstrated in the example sentence

*Dispersal of profilin_*A *_from such sites suggests that profilin_*B *_is involved in reorganization of actin cytoskeleton*.

where the entities *profilin*_*A*_, *profilin*_*B*_, *actin*, *actin cytoskeleton*, and *reorganization of actin cytoskeleton*would be annotated. Since the process *dispersal of profilin*_*A *_does not participate in any annotated relationship, it is not annotated. The protein *actin*, on the other hand, is nested in the entity *actin cytoskeleton*, which is in turn nested in the entity *reorganization of actin cytoskeleton*, the broadest entity relevant to the stated relationship *PARTICIPATE*(*profilin*_*B*_, *reorganization of actin cytoskeleton*).

#### Relationship and entity type ontologies

Before discussing the BioInfer relationship ontology, we briefly characterize its design goals in relation to those of other ontologies. There are a number of biomedical and top-level ontologies, each of which defines a part of the domain of the entities and relationships identified in BioInfer. For instance, many physical relationships on the molecular level are specified within the *molecular function *ontology of Gene Ontology (GO) [18]. Similarly, control processes such as *upregulation *are defined in the *biological process *ontology of GO. On the other hand, non-process relationships such as *sequence similarity *fall in scope of BioInfer annotation, but are not defined in GO. Moreover, BioInfer captures generic relationships such as *condition *whereby unknown molecular dependencies result in a process or event being a necessary condition for another process or event to take place. For instance in the sentence

Alpha-catenin links beta-catenin to the actin-based cytoskeleton

*Alpha-catenin *causes an underspecified relationship between *beta-catenin *and the *actin-based cytoskeleton*. However, the molecular basis for these relationships remain unspecified. There are no terms corresponding to the *cause/link *relationships at this level of generality in GO.

Another consideration is the aim of the BioInfer corpus at supporting IE systems in the domain. While pathway ontologies such as BioPAX [19] aim at the description of known molecular pathways, BioInfer aims at capturing what was stated in free text, which is usually not definite knowledge. The BioInfer relationship ontology captures the domain of statements about relationships rather than the relationships themselves. Thus, by using the ontology, the annotation avoids inference which refines text statements into pathway knowledge, and captures only the explicitly stated facts. Taking into account the considerations discussed above, we have designed the ontology to capture the relationships present in the BioInfer corpus text at a level of detail suitable for both the annotators and later application of the corpus to IE system development. The ontology was constructed based on the corpus data, to cover statements about the biologically relevant relationships found in the corpus text. It by enumeration defines the set of relationships relevant with respect to BioInfer. In this respect, it is closer to a hierarchical lexicon rather than a complete ontology defining the broad domain of biomedical entity interactions and relationships.

**Figure 3:**
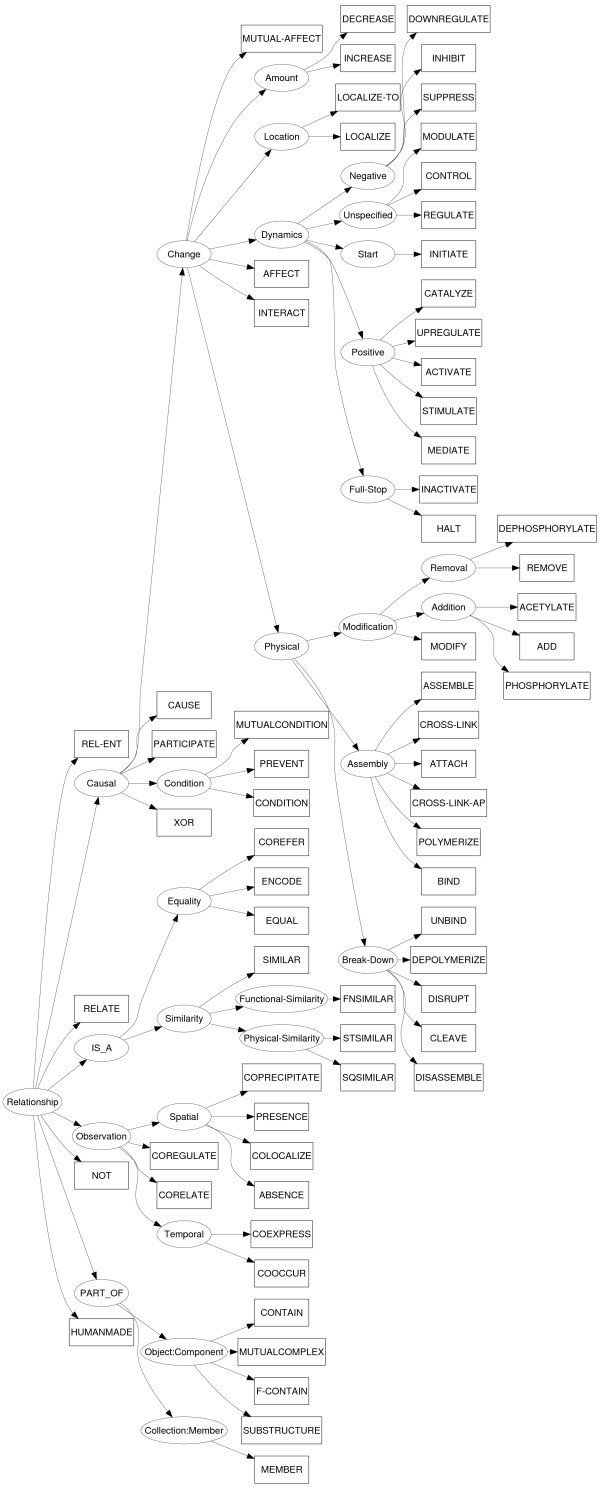
**The relationship type ontology**. Non-leaf nodes, drawn as boxes, are relationship type classes. Leaf nodes, drawn as ovals, are the predicate names used in the relationship annotation.

**Figure 4:**
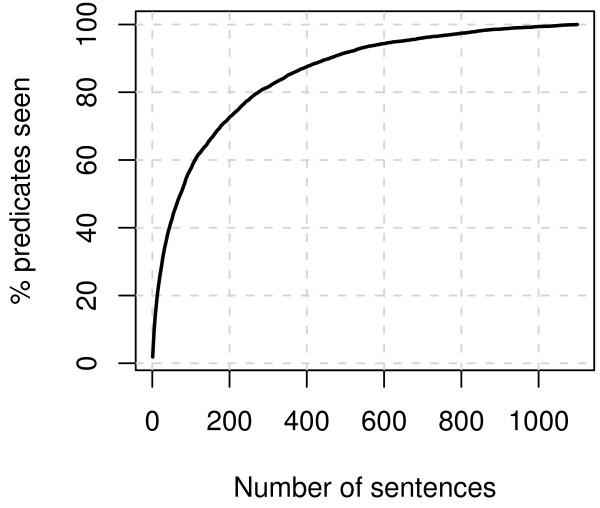
**Cumulative total predicate number**. The average cumulative total number of predicates seen, as fraction of all predicates, plotted against the number of sentences seen. The data for the plot is taken as the average over random orderings of the corpus sentences.

The relationship type ontology (Figure 3) specifies four broad classes of relationships. The *observation *class captures experimental observations, such as *co-occurrence *and *coprecipitation*. The classes *part*_- _*of *and *is*_-_*a *describe taxonomical, substructure/superstructure, physical/functional similarity, and equality relationships. The fourth class of relationships specifies *causal *relationships, where one entity causes a change in the state of another entity. While the class structure of the relationship ontology is fixed, the predicates are introduced into the ontology according to need – the choice of predicates is thus empirical and depends on the coverage of the annotated corpus. For example, of all the possible post-translational modifications only few, such as *PHOSPHORYLATE*, were sufficiently common in the corpus to merit a separate predicate in the ontology. To illustrate the coverage of the predicates of the annotated relationships and to estimate how often new predicates would need to be added in extending the annotation, we calculated the cumulative total number of different predicates that occur in the annotation when traversing the sentences in an arbitrary order (Figure 4). We find that, for example, 50% of all predicates are, on average, seen already after the first 74 sentences. Further, the total number of predicates seen increases only by one in approximately the last 220 sentences. We may extrapolate that to annotate another 1100 sentences, only approximately five new predicates would need to be introduced into the ontology.

The state of an entity, for the purpose of the BioInfer corpus, is characterized by four properties: *amount, location, dynamics*, and *physical*. These properties specify the amount, spatial distribution, degree of activity, and physical state of the entity. In the relationship type ontology, the *change *node, a specialization of the *causal *node, has for each of the four properties a subtree that contains predicates for relationships that affect the respective property, thus resulting in a change of the state. For example, an upregulation relationship affects the dynamics of an entity and therefore the *UPREGULATE *predicate is located in the *dynamics *subtree.

The Genia event ontology [20] serves a similar purpose as the BioInfer relationship ontology. It will be used in the future event annotation of the Genia corpus and has been designed as a modified subset of 34 terms from GO *biological process *(32 terms) and *molecular function *(2 terms) ontologies, with three additional terms. There are similarities in the structure of the BioInfer relationship and Genia event ontologies. For example, the *metabolism *vs. *regulation *distinction in the Genia event ontology is comparable to the *physical *vs. *dynamics *distinction in the BioInfer relationship ontology. However, the Genia event ontology focuses on the *metabolism *subtree, while the BioInfer relationship ontology focuses on the *dynamics *subtree, both ontologies providing a more detailed resolution at their focus point. Additionally, the Genia event ontology does not currently support non-process relationships.

Several groups have undertaken efforts to define and use *frames*, or conceptual structures, mostly focusing on the definition of predicate-argument structures for verbs [21-24]. Frame recognition has been proposed as a step in information extraction processes. In the biomedical domain a recent effort, PASBio, defined argument structures for 30 verbs known to be used to describe biological events [25]. PASBio takes a verb-oriented approach following the PropBank scheme. The BioInfer relationship annotation differs from efforts to define and use verb frames for IE in both aims and focus. However, we note that in this context the BioInfer approach most resembles FrameNet in that the annotation is centered around relationships instead of verbs and includes relationships stated through words other than verbs. Also, by contrast to PropBank and PASBio, BioInfer defines only the minimal core arguments of the relationship specifying the participants in the relationship.

Before we introduce the entity type ontology, let us consider an important contrast:

(i) *entity*_*A *_*causes entity*_*B *_*dephosphorylation*

(ii) *entity*_*A *_*dephosphorylates entity*_*B*_

(iii) *entity*_*A *_*inhibits entity*_*B *_*dephosphorylation*

There are two possible ways to annotate the sentence (i):

(a) *CAUSE*(*entity*_*A*_, *entity*_*B *_*dephosphorylation*)

(b) *DEPHOSPHORYLATE*(*entity*_*A*_, *entity*_*B*_)

The annotation (b) differs from the annotation (a) by interpreting the verb *cause*. By contrast, the sentences (ii) and (iii) have each only one possible annotation in the BioInfer corpus: the sentence (ii) would be annotated using (b) and the sentence (iii) would be annotated using (a) with *CAUSE *replaced by *SUPPRESS*. The sentences (i) and (iii) have the same surface structure and it would thus be practical if their annotation formulas had the same structure. On the other hand, sentences (i) and (ii) have a very similar (although not fully equal) meaning and it would thus be desirable for them to have the same annotation. Generally, this situation arises with generic statements such as *cause, result in*, and *lead to*. We chose not to interpret such generic statements. The sentence (i) would thus be annotated according to (a). To alleviate the impact of the decision on the contrast of (i) with (ii), we design the entity type ontology to mirror parts of the relationship type ontology in a way that allows automatic reinterpretation of the annotation from (a) to (b). We now introduce the entity type ontology.

**Figure 5:**
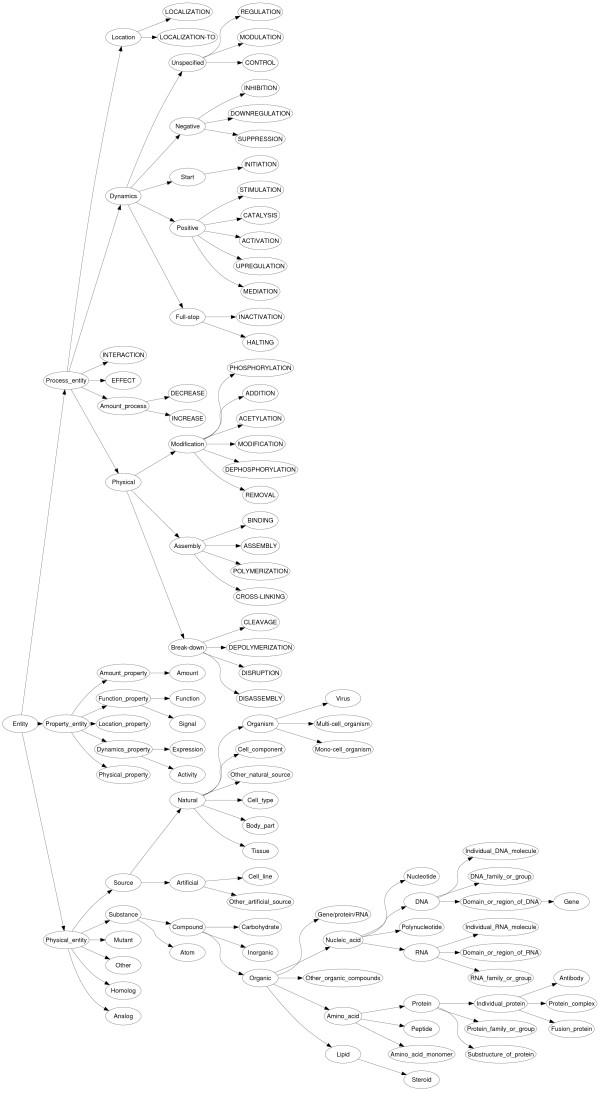
**The entity type ontology**. By contrast with the relationship type ontology, all nodes are used in the annotation. Note that the capitalized types have corresponding predicates in the relationship type ontology.

The entity type ontology (Figure 5) comprises of three main subtrees. The *physical entity *subtree is, up to minor differences, the Genia ontology of physical entities. For example, we introduce *gene *as a specialization of *domain or region of DNA *and *fusion protein *as a specialization of *individual protein*. The *process *subtree mirrors the *change *subtree in the relationship type ontology. For almost every state change in the *change *subtree of the relationship ontology (e.g. *phosphorylate*) there is a corresponding process in the *process *subtree of the entity type ontology (e.g. *phosphorylation*). The predicates in the *change *subtree of the relationship type ontology are used in sentences like (ii) while their corresponding process entity types are used in sentences like (i) and (iii). The last main subtree of the entity type ontology is the *property *subtree, which defines the main properties associated with entity state, that is, *amount, location, function, dynamics*, and *physical state*.

**Figure 6:**
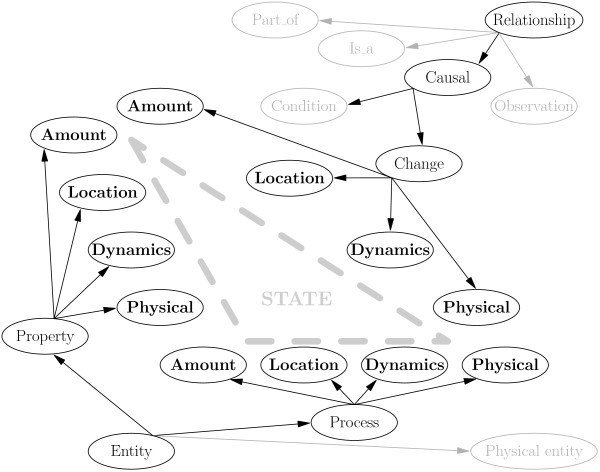
**The interrelationship of the ontologies**. The interrelationship of the ontologies is based on the notion of the *state *of an entity, characterized by the four properties: *amount, location, dynamics*, and *physical*. This division into four properties is found in three subtrees of the two ontologies: the *property *and *process *subtrees of the entity type ontology and the *change *subtree of the relationship ontology.

The interrelationship of the ontologies is illustrated in Figure 6. The key relationship between the ontologies is the correspondence of the *process *and *change *subtrees. This correspondence is due to the practical focus of the corpus on IE. In isolation, process extended entities are not of relevance, because they do not specify a relationship of several named bioentities. For instance, *cofilin phosphorylation *describes a biological process which is not of interest as a relationship *PHOSPHORYLATE*(_, *cofilin*), because its agent is left unspecified. However, it is of interest as a process if it participates in some other relationship. For this reason, we maintain separate definitions for the same underlying biological phenomenon as a relationship and as a process extended named entity. However, due to the correspondence between the ontologies, it is possible to automatically reinterpret the process extended named entities in terms of underspecified relationships, as exemplified in this paragraph, and thus remove the distinction.

#### Text binding

We have so far introduced the entities and relationships without discussing their binding to the text in the corpus. For instance, in the sentence

*Inhibition of actin_*A *_polymerization by a synthetic dodecapeptide patterned on the sequence around the actin_*B*_-binding site of cofilin*.

the desired output of an IE system is the *BIND*(*cofilin*, *actin*) relationship. While the exact words in the sentence which were used to extract this information, in this case *actin*_*A *_vs. *actin*_*B*_, are not necessarily of particular interest to the users of the IE system, they are of crucial importance in its development. It is important to specify text binding for the entities and relationships since correct information extracted from incorrect words is usually considered incorrect in IE system evaluation. In the example above, if the IE system used the word *actin*_*A *_to extract the relationship *BIND*(*cofilin*, *actin*), the output would have to be considered erroneous.

Up to very few exceptions detailed later in the Methods section, each entity and each predicate in the BioInfer corpus annotation is bound to the sentence text. As a rule, an entity is always bound to the minimal text that suffices to resolve the identity of the entity and its type within the ontology. A predicate is bound to the minimal text that suffices to infer the predicate name and thus the type of the corresponding relationship. Typically, assigning the text binding for an entity is straightforward and clear rules can be constructed to guide the annotators.

The text binding for a predicate is, on the other hand, less straightforward and developing a set of rules to guide the annotators is more difficult. Most relationships are stated with a verb and this verb then becomes the text binding for the predicate. There are, however, many other word and phrase categories that appear in a text binding for a predicate, such as *also known as *stating name equality and *recruitment of ... to *stating a localization relationship. The choice of the predicate based on the sentence text is context-dependent: there is no one-to-one correspondence between the words and phrases of the text and the predicates used. The relationship type ontology serves as a controlled vocabulary: it defines a set of types, each of which can be stated in many different ways in the text. For example, all of the phrases *affinity for, bind to, associate with, cofactor, contact to, contains site for, epitopes on, receptor for*, and many other, have been used in the corpus in text binding for the predicate *BIND*. On the other hand, the phrase *associate with *is also commonly bound to the predicate *RELATE *in cases where it does not imply direct physical binding. There is thus a many-to-many correspondence between the actual text phrases and the relationship types.

For the purpose of the dependency annotation, the sentence text is divided into tokens following a simple set of rules that can be easily implemented. However, for the entity and relationship annotation the tokens are not sufficiently detailed. Consider, for example, the token *Arp2/3 *which contains two entities, *Arp2 *and *Arp3*. We aim at capturing such entities and consequently our entity annotation scheme allows the tokens to be divided into sub-tokens. In the example, the token *Arp2/3 *is divided into the subtokens *Arp 2 / 3*. These subtokens are then combined into two entities, *Arp2 *and *Arp3*. The text correspondence of an entity is thus defined as a set of subtokens that, when textually concatenated, form the string identifying the entity. For nested entities, the subtokens of the inner entity are a proper subset of the subtokens of the outer entity.

Let us now summarize the key concepts in the entity relationship annotation. Relationships hold between entities. Entities can be either physical entities such as proteins or abstract entities such as processes and properties. The entities are nested to capture their inner structure. The type of each entity is annotated using the entity type ontology. The relationships among the entities are stated as logic formulas with predicates defined in the relationship type ontology, which also specifies the meaning of each predicate and the roles of its arguments. The annotation is bound to the text through sets of subtokens assigned to the entities and predicates. The ontologies are interdependent with the connection being the notion of entity state, state properties, and changes in state.

#### Dependency annotation

Many of the relationship statements are syntactically complex, involving, for example, coordination and long-distance dependencies between the words stating the relationship. To extract such relationships, many recently proposed IE systems employ full parsing [26-30]. To develop and evaluate both the parsing and domain analysis components of such systems, syntactic annotation is necessary.

The BioInfer corpus contains syntactic annotation using the Link Grammar (LG) formalism. LG is a well-documented dependency-type grammatical formalism with the advantage that the Link Grammar Parser is freely available and open source [31]. The LG grammar and its documentation can thus be examined in detail and serve as a reference for both annotators and users of the corpus. LG has further been applied to several BioNLP tasks, including IE targeting protein-protein interactions [27,29,32,33]. In producing the BioInfer corpus annotation, the LG formalism was followed systematically and extended only when necessary to provide annotation for phenomena not covered by the current version of the grammar. Due to the large number of dependency types in the LG formalism (approximately 100 main types and 400 types in total), we use an automatic method to assign the types; the method is presented in the Methods section.

In addition to considering correctness and completeness requirements in creating the annotation, we also take into account practical aspects of IE system development and adopt some of the approximations that are implemented in parsers that help to increase robustness and decrease ambiguity, while not being harmful to IE performance. For example, most parsers do not fully resolve noun phrase (NP) bracketing. As noun phrase internal structure is not a key issue for IE systems, we find the approximation acceptable and do not fully resolve NP bracketing. In contrast, we resolve semantically important ambiguities in the text such as prepositional phrase attachment, coordination, and relative clause attachment.

To extend the applicability of the dependency annotation we introduce *macro-dependencies*, a special form of annotation that can be expanded in different ways corresponding to the different, yet equally plausible, dependency analyses of a single grammatical phenomenon. Macro-dependencies thus allow systematic differences between dependency annotation schemes to be taken into account. We define *NP macro-dependency *to address the possibly most frequent systematic difference between dependency formalisms, the parallel vs. serial (chained) attachment of pre-modifiers to a noun. The choice of attachment scheme is largely arbitrary, with, for example, LG and MiniPar [34] attaching the pre-modifiers in parallel, and, for example, Connexor Machinese Syntax [35] attaching the pre-modifiers serially. Each parser systematically follows one of these two schemes instead of attempting to fully resolve the NP structure.

**Figure 7:**
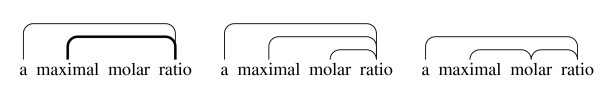
**An expansion of a macro-dependency**. Noun phrase with an NP macro-dependency (left), parallel expansion (middle) and serial expansion (right). NP macro-dependencies are depicted as thick lines.

In the annotation, an NP macro-dependency is used to connect the leftmost pre-modifier and the head noun. An NP macro-dependency can be expanded automatically to attach all pre-modifiers spanned by the macro-dependency in either of the two manners introduced above, making the annotation applicable to the two most common analyses (see Figure 7). Further, the macro-dependency is used consistently also in cases where there is only one pre-modifier. This allows the mere presence of the macro-dependency to be used as an indicator of a non-elementary noun phrase.

Macro-dependency annotation can be defined for many other grammatical phenomena where parsers systematically differ. However, annotation with many different types of macro-dependencies would increase the complexity of the annotation process and would require greater efforts from the annotators. For these reasons, we currently only apply NP macro-dependencies.

### Corpus statistics

The relationship annotation identifies 2662 relationships, of these 266 (10%) are of complex type, that is, one of their arguments is another relationship. Note that the negation predicate *NOT *is not counted as a complex relationship. Of the 2662 relationships, 163 (6%) are negated using the *NOT *predicate. The distribution of relationship types in the four top-level subtrees of the relationship type ontology is as follows: 1461 (55%) *causal*, 372 (14%) *is_a*, 575 (22%) *part_of*, 145 (5%) *observation*. Further 109 (4%) of the relationships belong to the most general *relationship *type. Of the 1100 sentences, 260 (24%) do not contain any relationship.

The entity annotation identifies 6349 entities. There are 4601 (72%) top-level entities, that is, entities that are not nested within any other entity. Of the 4601 top-level entities, 3318 do not contain any nested entity and 1283 do. Thus, fully 28% of top-level entities in the BioInfer corpus are complex, nested entities. Of the total 6349 entities, 4573 (72%) are named entities.

The corpus contains a total of 33858 tokens (29629 excluding punctuation). The average sentence length is thus relatively high, over 30 tokens. When NP macro-dependencies are expanded, the corpus contains 28139 word-to-word dependencies. Excluding punctuation, the annotation covers approximately 94% of all words, the most frequent unannotated words appearing in citations. There are notably many complex noun phrases and coordinations in the BioInfer corpus, as evidenced by the most commonly occurring dependency types: dependencies connecting noun pre-modifiers to nouns constitute approximately 21% of corpus dependency types and coordinations another 9%.

#### Quality of the annotation

As also discussed in the Methods section below, several steps were taken to assure the quality of the annotation, including redundant dependency annotation, frequent discussions among annotators to resolve and document ambiguous cases, and repeated verification of the annotated data against a set of written rules. These rules were formulated iteratively, from an initial tentative version to their final form documented in the annotation manual [36]. For the current initial release of the corpus, we did not undertake the effort to measure inter-annotator agreement, a measure of the stability of the annotation scheme. Rather, we have focused our limited resources on annotating as many sentences as possible while mainitaining the quality standard. Nevertheless, quantifying the reliablility of the annotation is an important issue that we intend to address in the future. We discuss below some of the issues relating to the measurement of inter-annotator agreement for the various types of annotation present in the corpus. The standard approach to measuring inter-annotator agreement on categorization tasks is to fully annotate a set of sentences by two separate annotators and calculate the *kappa statistic *[37,38], defined as κ=P(a)−P(e)1−P(e)
 MathType@MTEF@5@5@+=feaafiart1ev1aaatCvAUfKttLearuWrP9MDH5MBPbIqV92AaeXatLxBI9gBaebbnrfifHhDYfgasaacH8akY=wiFfYdH8Gipec8Eeeu0xXdbba9frFj0=OqFfea0dXdd9vqai=hGuQ8kuc9pgc9s8qqaq=dirpe0xb9q8qiLsFr0=vr0=vr0dc8meaabaqaciaacaGaaeqabaqabeGadaaakeaaiiGacqWF6oWAcqGH9aqpdaWcaaqaaiabdcfaqjabcIcaOiabdggaHjabcMcaPiabgkHiTiabdcfaqjabcIcaOiabdwgaLjabcMcaPaqaaiabigdaXiabgkHiTiabdcfaqjabcIcaOiabdwgaLjabcMcaPaaaaaa@3EC7@, where *P*(*a*) is the measured probability of agreement between annotators, and *P*(*e*) is the probability that agreement is due to chance. As the BioInfer corpus contains several types of annotation, each of which is to some extent independent of the others, it is natural to measure agreement separately for the different annotation types. However, even with this simplification, there are a number of difficulties in calculating the *κ *statistic for many of the annotation types, including the following:

• If may be difficult or impossible to calculate *κ *when the set of possible annotations is very large or not clearly defined [39]. For example, the space of possible entity occurrences, potentially discontinuous and overlapping, is huge, causing simple estimates of *P*(*a*) to approach one and *P*(*e*) to approach zero: annotators will agree that the vast majority of possible entities should not be annotated. Similar issues arise in relationship annotation and, to some extent, also in dependency annotation.

• In calculating *κ*, the annotation categories are assumed to be discrete and mutually exclusive, so that each categorization is either correct or absolutely incorrect. However, as the entity and relationship types are hierarchical, it would be appropriate to recognize different degrees of correctness in type categorization, for example when one annotator assigns a specific and another a related, but less specific type to an entity.

• The *κ *statistic essentially measures adjusted accuracy, which is not the favored measure of performance for many of the annotation types in the corpus. For example, for entity recognition, separate measurement of *exact *and *sloppy *matches [32] or *boundary matches *[40] could be more informative.

For these reasons we intend, instead of using *κ*, to measure agreement separately for the different annotation types, applying the most informative measures for each type.

### Related biomedical corpora

Cohen et al. maintain a public resource [41] that collects information about existing biomedical corpora, including those discussed in their recent corpus comparison [42]. In addition, Hakenberg maintains a similar collection [43]. These two collections together comprise twenty corpora that are primarily intended for biomedical NLP. These corpora differ substantially as to their annotation types, dataset sizes as well as usage rates. In the following, we use the names from these two collections when referring to other existing corpora.

While most of the twenty corpora provide named entity annotation of some kind, the de facto standard for biomedical named entity recognition research is the Genia corpus [8]. It provides annotation for all biomedical entities in the text, as compared to genes, proteins, RNAs and related in the BioInfer corpus. The Genia annotated entities are physical entities, that is, abstract entities such as properties and processes are not annotated. The entities are given types from the Genia ontology which forms a part of the entity type ontology used in the BioInfer corpus.

Syntactic annotation is provided by five corpora (BioIE, Brown-Genia treebank, Genia treebank, DepGenia, LLL). The former three provide constituency annotation, the latter two provide dependency annotation, where for DepGenia, the annotation is obtained automatically. With the recent interest in dependency parsers and their application to various NLP tasks, the dependency annotated corpora complement the constituency annotated corpora.

Relationship annotation is provided by six corpora (BioText, IEPA, PDG, Wisconsin, LLL, BC). For BioText, the related entities are disease and treatment, the other five contain protein-protein interactions and, in several cases, other entity relationships such as gene-disease or protein-location. In all cases, the relationships are pairwise.

There are two aspects that set the BioInfer corpus apart in comparison with the corpora listed above. The first aspect is the relationship annotation that captures also complex non-binary relationships and classifies these in a large number of hierarchically ordered relationship types. To our knowledge, no other biomedical corpus provides such a detailed annotation of entity relationships. The second aspect is the combination of the different annotation types for the main steps in common IE systems. As discussed in the Background section, the availability of all these annotation types for a single set of sentences gives, for example, an opportunity to study the relationship and error propagation in the components of IE systems rather than only develop and evaluate the components in isolation. To our knowledge, the combination of named entity, syntactic, and relationship annotation is provided, in addition to BioInfer, only by the LLL corpus. The LLL corpus is, however, much smaller than the BioInfer corpus, consisting of 166 sentences, and contains only untyped binary directed relationships. Many types of relationships annotated in BioInfer are thus not annotated in LLL.

On the other hand, when comparing the individual annotation types to other corpora, it is natural that a specialized corpus provides a more detailed individual annotation than the BioInfer corpus. For instance, the Genia physical entity resolution is more detailed and coverage broader than the physical entity annotation in the BioInfer corpus.

## Conclusion

In this paper, we have introduced a resource aimed at developing IE systems and their components in the biomedical domain. We have developed a scheme providing the key types of annotation for a single set of sentences, expressing complex relationships between both physical and abstract entities. As part of the annotation scheme, we have introduced two ontologies defining the types of entities and their relationships. Using this annotation scheme, we have developed a corpus of 1100 sentences containing full dependency annotation, dependency types and comprehensive annotation of bioentities and their relationships. The BioInfer corpus is publicly available [17].

As future work, we intend to apply the corpus in the development and evaluation of an IE system and in the process identify the strengths and weaknesses of the current annotation. Based on this experience, we plan to enhance and extend the corpus annotation to further increase its utility as a resource for biomedical natural language processing.

## Methods

In this section, we present in greater detail the use of special predicates for, e.g., coreference and abbreviations, and discuss the rules determining the textual extent and type of annotated entities. A comprehensive collection of annotation rules regarding entities, relationships and their types is given in the BioInfer annotation manual [36].

Further, we present the dependency annotation of coordination and the method used to automatically determine dependency types. Finally, we discuss the source of the corpus text and the annotation process.

### Coreference

Let us consider the following sentence.

*Cadherins are essential for morphogenesis since they can modulate beta-catenin signaling*.

The only relevant relationship in the sentence is *CONTROL*(*cadherins*, *beta-catenin signaling*). Apart from the relationship itself, it is also crucial to consider how an IE system would extract the relationship, and provide corresponding annotation in the corpus. In the sentence above, many IE systems would resolve the coreference between *cadherins *and the pronoun *they *and extract the relationship through the pronoun which is syntactically closer to the relationship statement.

In the BioInfer corpus, coreference is annotated using the predicate *COREFER*. The annotation for the sentence above would thus be *COREFER*(*they*, *cadherins*) and *CONTROL*(*they*, *beta-catenin signaling*). The *COREFER *predicate captures an asymmetrical relationship where the first argument is semantically dependent on the second argument. The predicate is interpreted as a simple textual replacement of the text of the first argument with the text of the second argument. In the majority of cases, the second argument of *COREFER *is a named biomedical entity. There are, however, cases where this is not true. Consider, for instance, the sentence

*Gamma-catenin distribution is remarkably similar to that of beta-catenin*.

where the annotation contains *COREFER*(*that*, *distribution*). Under the textual-replacement interpretation, *that *is replaced with *distribution *to obtain the entity *distribution of beta-catenin*. Coreference is resolved only in cases where the resolution is necessary for annotating a relevant relationship. The exact rules governing the usage of the *COREFER *predicate are further detailed in the annotation manual.

### Implicit reference

In the sentence

*The addition of profilin to actin filaments causes slow depolymerization*.

the relationships are *CAUSE*(*ATTACH*(*profilin*, *actin filaments*), *depolymerization of actin filaments*). These relationships cannot be directly annotated because the sentence states only implicitly that *depolymerization *relates to *actin filaments*. This case can, however, be viewed as a case of nesting with its inner-most element not realized. We use the predicate *REL-ENT *to state the implicit reference. The annotation for the example sentence would be

*CAUSE*(*ATTACH*(*profilin*, *actin filaments*), *depolymerization*) and *REL-ENT*(*depolymerization*, *actin filaments*). The *REL-ENT *predicate is interpreted as a nesting, equivalent to the use of the phrase *depolymerization of actin filaments*.

### Abbreviation definitions and entity equality

The *EQUAL *predicate captures the equality of two entities. The use of the *EQUAL *predicate is illustrated in the examples below. The most common case of equality is abbreviation and synonym definition (i-ii), other cases are statements of the type *X is Y *(iii), and statements with several names for the same bioentity (iv).

(i) the expression of E-cadherin (E-Cad) → *EQUAL*(*E-cadherin*, *E-Cad*)

(ii) MORT1 (also called FADD) → *EQUAL*(*MORT1*, *FADD*)

(iii) MDP2 is the previously identified VRP1 → *EQUAL*(*MDP2*, *VRP1*)

(iv) cofilin/ADF is an important regulator → *EQUAL*(*cofilin*, *ADF*)

Semantically the *EQUAL *predicate is symmetrical, that is, *EQUAL*(*a*, *b*) is interpreted identically to *EQUAL*(*b*, *a*). The predicate is interpreted such that when *EQUAL*(*a*, *b*), any relationship that holds for *a *holds also for *b *and vice versa.

### Statements of confidence and negation

Different levels of confidence or conclusiveness of the reported results are currently not annotated. Relationships introduced through statements such as *we suggest that *or *we tested for *are annotated as if the statements were absent. Statements on the absence of a relationship such as *not affected by *or *independent of *are annotated using a special predicate *NOT*.

### Anonymous entities

Rarely, an entity may have no realization in the text (0.3% of all entities). Such entities are called *anonymous entities *and have a type but no text binding. Anonymous entities most commonly occur with statements of protein complex forming such as

*Cadherin molecules are complexed with alpha-catenin and beta-catenin*.

In accordance with the BioInfer annotation of protein complexes, the complex is an entity whose constituents are related to it through the *CONTAIN *relationship. In the example above, the complex is an anonymous entity. The annotation of the sentence is as follows

*ANONYMOUS*(*X*), *CONTAIN*(*X*, *cadherin molecules*), *CONTAIN*(*X*, *alpha-catenin*), *CONTAIN*(*X*, *beta-catenin*)

### Entities outside the annotation scope

As a general principle, only those entities that have a named gene, protein, or RNA at their innermost level of nesting are annotated. There are, however, complex relationships where, for example, a protein affects a relationship of another protein with an entity falling outside the scope of the annotation. Consider the sentence

*Profilin inhibits hydrolysis of PIP2 by phospholipase C*.

with the relationship *SUPPRESS*(*profilin*, *CLEAVE*(*phospholipase C*, *PIP2*)). Since *PIP2 *falls outside the annotation scope, no relationship could be annotated. In cases of the general type *REL*(*X*, *REL*(*Y*, *Z*)) where *Y *or *Z *falls outside the annotation scope, we make an exception and annotate the entity in order to capture the relationship of the proteins. Such an annotation is marked with a special predicate *OTHER *and can thus be disregarded for some applications of the corpus.

### Entity extent and typing

Established names of genes, proteins, and RNA as well as functionally well-defined gene/protein families and groups are annotated without nesting, so that when a named entity contains other candidate named entities, only the broader, contextually salient entity is annotated. For example, as *cyclin-dependent kinase inhibitors *is an established protein family name, it is annotated as a single name even though it contains the candidate names *cyclin *and *kinase*.

The extent of named entities also includes the source within an organism, such as tissue or organ, as they appear in established names. For example, *alpha-smooth muscle actin *is annotated as a whole, instead of, for example, *alpha-actin *or only *actin*. By contrast, such specifications appearing as prepositional phrases or relative clauses are not considered part of the name: in *actin from rabbit skeletal muscle, actin *is annotated as the name. Organism names are not considered part of gene/protein/RNA names, but are included in extended named entities: in *Acanthamoeba profilin inhibits Acanthamoeba actin polymerization*, the basic named entities are *profilin *and *actin*; the organism *Acanthamoeba *is part of the extended named entities only.

In determining the extent of extended named entities, the general rule is to include only the minimum necessary to include the named entity the extended named entity pertains to and resolve the type of the extended named entity. Thus, for example in *TD77 disrupts actin stress fibers*, the related entities are *TD77 *and *actin fibers*, the latter excluding the modifier *stress*. Likewise, in *cofilin plays a crucial role in rapid remodeling of the cortical actin meshwork *the entities are *cofilin *and *remodeling of actin meshwork*, excluding *rapid *and *cortical*. This approach allows differentiating key phrases stating entities from modifiers appearing only coincidentally. Note that the attachment of the omitted modifiers to the entity is still preserved by the dependency annotation.

Entity types are assigned from the entity type ontology to all annotated entities. In typing named entities (72% of all entities), we took advantage of the relationship annotation to automatically assign types to those named entities whose type was determined by the relationships they participate in (17% of all entities). Additionally, a number of named entities were assigned types based on their names when the annotators decided that the name unambiguously describes a specific type (25% of all entities). For entities that are not involved in any relationship nor nested in entities that are, i.e. entities that are not relevant to any relationship, only the generic type *gene/protein/RNA *is currently assigned (19% of all entities). The remaining untyped named entities (10% of all entities) as well as all entities which are not named entities (28% of all entities), were assigned their type by manual examination (38% of all entities).

### Dependency annotation for coordination

**Figure 8:**
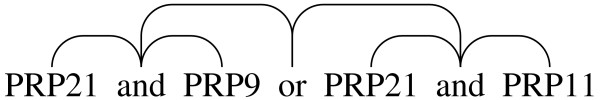
**Layered coordination annotated using the LG approach**. Coordinations with *and *as constituents in a coordination with *or*.

**Figure 9:**
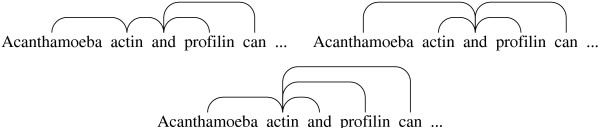
**Interpretation of coordination**. LG annotation of the coordination [[*Acanthamoeba actin*] *and *[*profilin*]] (top left) and [*Acanthamoeba *[*actin and profilin*]] (top right). Chained annotation (bottom) is not capable of expressing the distinction and the annotation is thus the same for both cases.

Capturing coordination in the dependency syntax framework is not straightforward and there exist various approaches to the problem. The Link Grammar and, for example, the PARC 700 Dependency Bank annotation [44], capture coordination using a structure in which the coordinated elements are dependents of the coordinator, which in turn represents the functional role of each of the coordinates. In contrast, the Connexor Machinese Syntax parser, for example, chains the coordinated elements and the head of the chain shows the functional role of the coordinated units, while the coordinator is a mere dependent of one of the elements in the chain. See Figures 8 and 9 for illustration of the structures discussed.

We follow the LG approach to coordination. The LG approach is more expressive than the chaining described above, allowing, for example, a single modifier to apply to all of the coordinated elements (Figure 9). The more expressive LG annotation can be transformed to the less expressive chaining annotation, but not vice versa. Further, we consider the fact that all of the coordinated elements occur at the same level in the parse structure to be an obvious advantage for IE system development.

**Figure 10:**
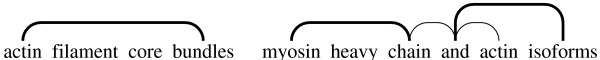
**NP bracketing**. Unresolved (left) vs. resolved at the level of coordination (right) NP bracketing. Thick lines denote NP macro-dependencies.

In a corpus that is used to develop tools for extracting bioentity relationships, it is not critical to resolve the internal structure for every noun phrase, a general principle followed by both BioInfer and, for example, the Genia treebank. In the BioInfer corpus, pre-modifier attachment in noun phrases without internal coordination is not resolved, as these phrases typically only contain at most one named entity. In noun phrases that contain internal coordination, however, each of the coordinates can represent a different named entity and resolving the coordination and thus separating the named entities from each other is vital to the IE task. The distinction is illustrated in Figure 10. From the perspective of an IE system extracting bioentity relationships, the difference between the correct bracketing [[*actin filament*] *core bundles*] and the unresolved bracketing [*actin filament core bundles*] is not critically important because *actin *is the only protein in the phrase and the inner structure of the phrase can be ignored. On the contrary, the correct resolution of the coordination [[[*myosin heavy chain*] and [*actin*]] *isoforms*] is important because the coordinates are protein names and hence of importance to the IE system. While the inner structure of the coordinated phrases is still not necessarily fully resolved, the two protein names are separated by the coordination. Further, while major wide-coverage dependency parsers such as Connexor Machinese Syntax, MiniPar, and Link Parser do not resolve the attachment of pre-modifiers in noun phrases, they do resolve coordination. The annotation in BioInfer thus coincides with the intended coverage of these parsers.

### Dependency types

During the manual dependency annotation, the dependency types were not specified. Due to the large number of dependency types defined in LG (more than 100 main types with almost 400 subtypes) the effort to manually determine the type of each dependency is prohibitive. However, dependency types are important for many uses of the corpus. Thus, we have developed a reliable heuristic method for determining dependency types automatically given the dependency structure. This method has been used to assign dependency types to the whole corpus. In addition, quality control of the dependency types has been performed by manual examination.

The method to determine dependency types has four main steps: reducing the complexity of the sentences, parsing the simplified sentences, assigning dependency types based on the parses, and extending the types to the full sentences using a set of rules. In the following, these steps are described in more detail.

#### Sentence simplification

NP macro-dependencies have an important role in the simplification of the sentences. As the attachment of pre-modifiers in noun phrases covered by NP macro-dependencies can be determined with simple rules without parsing, any NP macro-dependency present in the sentences can be truncated so that all the pre-modifiers spanned by the macro-dependency are removed. This procedure reduced the number of words by more than 20% and removed all problems related to the parsing of these noun phrases. The sentences were further simplified by capitalizing common uncapitalized proper names and removing citations.

#### Parsing

We parsed the simplified sentences with a modified version of the LG parser that overcomes some of the problems of the unmodified LG parser for biomedical text [10]. The modifications include an extended biomedical lexicon [33] as well as support for many of the grammatical phenomena not recognized by the unmodified parser. For each sentence, we consider up to a thousand alternative parses produced by the parser.

#### Majority types

For each sentence, we determine by comparison against the manually created dependency structure the subset of parses with the maximal number of recovered dependencies. The intuition underlying this selection heuristic is that parses having correct dependency structure also have the correct dependency types. From the set of maximally correct parses, we then determine for each dependency the majority type, that is, the type which most commonly appears for the dependency in the parses.

#### Finalization

We transfer the majority types from the simplified to the full sentences and resolve the types of the dependencies associated with the truncated noun phrases using straightforward rules.

We found that the quality of dependency types is significantly higher for sentences that receive fully correct parses in the parsing step. We thus mark sentences depending on whether or not they were fully correctly parsed, allowing users of the corpus to choose whether to use all dependency types or only the most reliable ones. To evaluate the quality of the automatically produced dependency types, we manually examined 30 randomly chosen sentences with fully correct parses and 30 randomly chosen sentences for which fully correct parses were not obtained.

For the fully correctly parsed sentences, the heuristic method assigned the correct dependency type to 575 out of 586 dependencies (98%). For the sentences without fully correct parse, 828 out of 962 dependencies (86%) were assigned their correct type. Out of the 1100 sentences, 741 (67%) received a fully correct parse.

### Corpus data

The sentences that form the corpus text were selected using the following procedure. Pairs of proteins that are known to interact were extracted from the Database of Interacting Proteins DIP [45,46]. These pairs were entered as search terms into the PubMed retrieval system and the returned publication abstracts including titles were split into sentences. These sentences were then searched for the protein pairs, giving a set of 2240 sentences that contain the names of at least two proteins that are known to interact (the selection was performed in December 2001). Compared to a random sample of PubMed, this selection procedure results in a corpus with a much higher proportion of relevant sentences, that is sentences that state actual relationships. The sentences are preserved as they appear in the article abstracts, including spelling errors, grammatical mistakes, and, for example, embedded citations.

### Supporting software

The BioInfer corpus is provided in an XML format. XML is a standard text-based format for structured documents, and XML parsers are freely available. Due to the relatively complex structure of some of the annotation types in the BioInfer corpus, we provide supporting software to further ease the use of the corpus.

**Figure 11:**
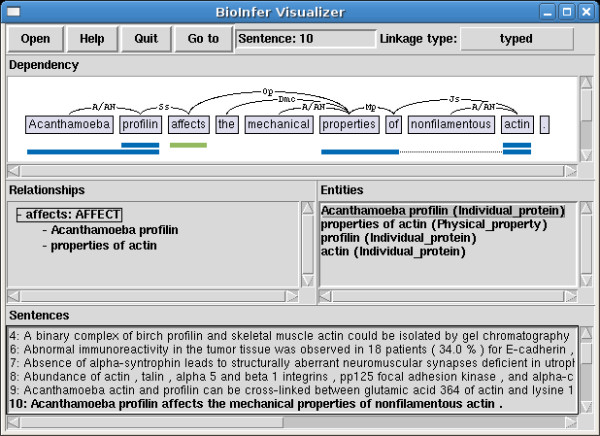
**The BioInfer visualizer**. BioInfer visualizer graphical user interface showing sentence with syntactic annotation with typed dependencies, with text corresponding to selected entities and relationships highlighted (topmost). A hierarchical view of relationships is shown middle left and entities with their types on the middle right. The bottom of the screen contains a sentence selector.

The supporting software is based on an extendable framework for parsing the corpus and representing it as data structures that can be accessed through a fully documented API (Application Programming Interface) providing methods for accessing different aspects of the annotation. In addition, we provide programs built on this API that visualize the annotations and extract them in a simplified, human-readable form (see Appendix I). The user interface of Bioinfer Visualizer is shown in Figure 11. With the BioInfer API, it is easy to create programs which, for example, analyze the corpus data or transform the annotation into formats required by other software. All supporting software is written in Python, thoroughly documented, and available under an open-source license on the corpus website.

### Annotation process

It is common practice to use automatically parsed text as the starting point for syntax annotation. However, since general English parsers could not provide sufficiently accurate parses due to their generally poor performance on biomedical text, we chose to create the dependency annotation manually without a baseline parse. Fully manual annotation also helps to avoid bias toward systematic errors introduced by the parser.

The dependency annotation was created by six annotators who worked in rotating pairs to reduce variation and avoid systematic errors. The set of sentences was divided into batches of nine sentences and each batch was assigned to one pair for annotation. For each batch the dependency annotation was first created by both members of the pair independently. The annotations were then compared and in case of differences, the matter was discussed until an agreement was reached. Two of the annotators were biology experts and the other four annotators had a possibility to consult an expert. Further, all annotators acquired thorough knowledge of the LG dependency formalism.

The entity and relationship annotations were created in parallel, partially based on a previous unpublished entity and relationship annotation of the corpus. This annotation was created by a biology expert, difficult cases and annotation rules being discussed with two IE researchers. The pair annotation strategy used in producing the dependency annotation was not applied for the entity and relationship annotation. The annotation was produced in several passes through the data as the rules and annotation types were refined, taking into account experience gained in the previous annotation. The last full pass was carried out shortly before publication.

We estimate that the annotation of the BioInfer corpus consumed 2500 man-hours, that is, 15 man-months, not including time spent on previous, unpublished annotation of the data, tool building, development of supporting software, and the design of the annotation scheme.

## Appendix I – Relationship annotation examples

In this Appendix, we list several annotated sentences from the corpus, in order to demonstrate the relationship annotation scheme on non-trivial, real-world cases. For each example, we list all annotated relationships along with other information when necessary.

Examples

**(a)*** Biochemical analyses revealed a strong induction of VEGF-receptor-2 (flk-1/KDR) tyrosine-autophosphorylation by VEGF which was maximal after 5 minutes and was followed by receptor down-regulation*.

*EQUAL*(*VEGF-receptor-2*, *flk-1*)

*EQUAL*(*VEGF-receptor-2*, *KDR*)

*induction of by:INITIATE*(*VEGF*, *tyrosine-autophosphorylation:PHOSPHORYLATE*(*VEGF-receptor-2*)

Autophosphorylation is represented with a one-argument predicate *PHOSPHORYLATE*.

**(b)*** In investigating the mechanism by which pRb induces senescence, we have found that pRb causes a post-transcriptional accumulation of the cyclin-dependent kinase inhibitor *<_*a*_*p27*_*a*_> (<_*b*_*KIP1*_*b*_, >) *that is accompanied by an increase in *<_*c*_*p27*_*c*_>(<_*d*_*KIP1*_*d*_>)* specifically bound to cyclin E and a concomitant decrease in cyclin E-associated kinase activity*.

*EQUAL*(<_*a*_*p27*_*a*_>, <_*b*_*KIP1*_*b*_>*)*

*EQUAL*(<_*c*_*p27*_*c*_>, <_*d*_*KIP1*_*d*_>)

*MEMBER*(*cyclin-dependent kinase inhibitor*, <_*a*_*p27*_*a*_>)

*causes:CAUSE*(*pRb*, *accumulation of p27*)

*bound to:BIND*(<_*c*_*p27*_*c*_>,*cyclin E)*

*accompanied by:COOCCUR*(*accumulation of p27*, *increase in p27*)

*accompanied by:COOCCUR*(*accumulation of p27*, *decrease in cyclin E-associated kinase activity*)

*concomitant:COOCCUR*(*increase in p27*, *decrease in cyclin E-associated kinase activity*)

**(c)*** Analysis of the formation of the calf spleen complex in the presence of varying concentrations of divalent cations gave evidence for the presence of a high-affinity divalent-cation-binding site on the spleen actin *(*beta*, *gamma*) *which appears to regulate the interaction with profilin*.

*regulate:CONTROL*(*site on spleen actin beta*, *interaction with:INTERACT*(*spleen actin beta, profilin*))

*regulate:CONTROL*(*site on spleen actin gamma*, *interaction with:INTERACT*(*spleen actin gamma, profilin*))

The stated interaction of *profilin *with the *spleen actins *is annotated directly as there are no coreferents for the proteins.

**(d)*** Neuropilin 1 (NP-1) is a receptor for vascular endothelial growth factor (VEGF) 165 (VEGF165) and acts as a coreceptor that enhances VEGF165 function through tyrosine kinase VEGF receptor 2 (VEGFR-2)*.

*EQUAL*(*neuropilin 1, NP-1*)

*EQUAL*(*vascular endothelial growth factor 165, VEGF165*)

*EQUAL*(*VEGF receptor 2, VEGFR-2*)

*receptor for:BIND*(*neuropilin 1, vascular endothelial growth factor 165*)

*MEMBER*(*tyrosine kinase, VEGF receptor 2*)

*through:MEDIATE*(*VEGF receptor 2, enchances:ACTIVATE*(*neuropilin 1, VEGF165 function*))

*through:RELATE*(*neuropilin 1, VEGF receptor 2*)

**(e)*** SNF11, a new component of the yeast SNF-SWI complex that interacts with a conserved region of SNF2*.

*component of:CONTAIN*(*SNF-SWI complex, SNF11*)

*complex: CONTAIN*(*SNF-SWI complex, SNF*)

*complex: CONTAIN*(*SNF-SWI complex, SWI*)

*interacts with:INTERACT*(*SNF11, region of SNF2*)

Since *SNF11 *and *component *are syntactically bound through a dependency that unambiguously identifies the coreference (see the annotation manual [36] for the relevant definition of syntactic binding), the interaction is annotated directly between *SNF11 *and *region of SNF2*.

**(f) ***Deletion of SIR4 enhanced mURA3 and MET15 silencing, but deletion of SIR1 or SIR3 did not affect silencing, indicating that the mechanism of silencing differs from that at telomeres and silent mating loci*.

*enhanced:STIMULATE*(*deletion of SIR4*, *mURA3 silencing*)

*enhanced:STIMULATE*(*deletion of SIR4*, *MET15 silencing*)

*COREFER*(*silencing, mURA3 silencing*)

*COREFER*(*silencing, MET15 silencing*)

*not:NOT*(*affect:AFFECT*(*deletion of SIR1*, *silencing*))

*not:NOT*(*affect:AFFECT*(*deletion of SIR3*, *silencing*))

Here, in contrast with the previous example, the coreference cannot be trivially recovered from the dependency and therefore it is annotated. Moreover, *silencing *acts as a coreferent for two silencing processes.

**(g)*** Discrete segments *(*70–150 amino acids*) *of PRT1 and TIF35 were found to be responsible for *<_*ab*_*their*_*ab*_> *binding to TIF34*.

*COREFER*(<_*a*_*their*_*a*_>,*PRT1*)

*COREFER*(<_*b*_*their*_*b*_>,*TIF35*)

*responsible for:CAUSE*(*segments of PRT1, binding to:BIND*(<_*a*_*their*_*a*_>,*TIF34*))

*responsible for:CAUSE*(*segments of TIF35, binding to:BIND*(<_*b*_*their*_*b*_>,*TIF34*))

There are two distinct proteins and a segment on each of the proteins, where each segment is responsible for the binding of its respective protein to *TIF34*. In order to capture these relationships accurately, there are two separate entities annotated that mark the pronoun their as a coreferent for the proteins.

**(h)*** Although talin has been suggested to act as a linkage protein mediating the attachment of <_*c*_<_*ab*_GP*_*ab*_><_*a*_*IIb*_*a*_>-<_*b*_*IIIa*_*b*_>_*c*_> *to actin filaments, direct binding of <_*f*_<_*de*_GP_*de*_><_*d*_IIb_*d*_>-<_*e*_IIIa_*e*_>_*f*_> to this cytoskeletal protein has not been demonstrated*.

*COREFER*(*this*, *talin*)

*mediating:MEDIATE*(*talin, attachment of:ATTACH*(<_*c*_*GPIIb-IIIa*_*c*_>, *actin filaments*))

*binding of to:BIND*(<_*f *_*GPIIb-IIIa*_*f *_>, *this protein*)

*CONTAIN*(<_*c*_*GPIIb-IIIa*_*c*_>, <_*a*_*GPIIb*_*a*_>)

*CONTAIN*(<_*c *_GPIIb-IIIa_*c *_>, <_*b *_GPIIIA_*b*_>)

*CONTAIN*(<_*f*_*GPIIb-IIIa*_*f *_>, <_*d*_*GPIIb*_*d *_>)

*CONTAIN*(<_*f*_*GPIIb-IIIa*_*f *_>, <_*e*_*GPIIIa*_*e*_>)

The coreference between *talin *and *this *is annotated and therefore, under the textual-replacement interpretation of coreference, the binding is with *talin protein*.

**(i)*** A 2.2-kb truncated NRP1 cDNA was cloned that encodes a 644*-*aa soluble NRP1 (sNRP1) isoform containing just the a/CUB and b/coagulation factor homology extracellular *<_*ab *_*domains of NRP1 *_*ab*_>.

*EQUAL*(*soluble NRP1*, *sNRP1*)

*encodes:ENCODE*(*truncated NRP1 cDNA, soluble NRP1 isoform*)

*containing:SUBSTRUCTURE*(*soluble NRP1 isoform*, <_*a*_* domains of NRP1*_*a*_>)

*containing:SUBSTRUCTURE*(*soluble NRP1 isoform*, <_*b *_*domains of NRP1*_*b*_>)

*homology:SIMILAR*(<_*a *_*domains of NRP1*_*a*_>,*CUB*)

*homology:SIMILAR*(<_*b *_*domains of NRP1*_*b*_>, *coagulation factor*)

The sentence states a relationship of a mutant gene *truncated NRP1 cDNA *rather than its wild-type form *NRP1 cDNA*. This is also reflected in the entity types, where *NRP1 cDNA *is given the type *gene*, while *truncated NRP1 cDNA *is given the type *mutant*.

**(j) ***CD26 is a T cell activation antigen known to bind adenosine deaminase and have dipeptidyl peptidase IV activity*.

*bind:BIND*(*CD26, adenosine deaminase*)

*have activity:FNSIMILAR*(*CD26, dipeptidyl peptidase IV*)

The statement of *CD26 *having *dipeptidyl peptidase IV *activity is interpreted as a functional similarity between the two proteins. Like other names that can refer to both a type of function and a distinct protein, *dipeptidyl peptidase IV *is considered a protein in BioInfer.

**(k)*** Data is presented to suggest that the G1 cyclin D1 and the cyclin-dependent kinase inhibitor p27KIP1 may be involved in subversion of the G1/S traverse by signaling pathways activated by HER-2 function*.

*MEMBER*(*cyclin-dependent kinase inhibitor*, *p27KIP1*)

*involved in:PARTICIPATE*(*cyclin D1*, *subversion of traverse by pathways activated by HER-2 function*)

*involved in:PARTICIPATE*(*p27KIP1*, *subversion of traverse by pathways activated by HER-2 function*)

The text *subversion of the G1/S traverse by signaling pathways activated by HER-2 function *gives rise to the entity nesting <*subversion of *<*traverse by *<*pathways activated by *< <*HER-2*> *function *> > > >

## Availability and requirements

**Project name: **BioInfer corpus and supporting software

**Project homepage: **

**Operating systems: **Platform independent

**Programming languages: **Python

**Other requirements: **Tkinter library for Python

**Licence: **LGPL

## Authors' contributions

SP and FG are the primary authors of this work and their contribution is equal. SP and FG have conceived the corpus annotation scheme in its present form, with a significant contribution by JH to the biomedical aspects of the annotation. The text of the paper was written by SP and FG. JH was the principal annotator for the entity and relationship annotation. The dependency annotation was produced jointly by JH, JBj, SP, and FG. The entity type annotation was produced by JH and JBj. The work was supervised by JBo, JJ, and TS since its beginning in late 2001, and they have contributed to both the content and the form of the corpus and the paper. All authors read and approved the final manuscript.
